# P04.26. Prevalence of botanical dietary supplement use among Hispanics in the United States: a systematic review

**DOI:** 10.1186/1472-6882-12-S1-P296

**Published:** 2012-06-12

**Authors:** P Gardiner, A Filippelli, K Faurot

**Affiliations:** 1 Department of Family Medicine, Boston University Medical Center, Boston, USA; 2 Department of Family Medicine, Boston Medical Center, Boston, USA; 3 UNC Epidemiology, Department of Physical Medicine & Rehabilitation, Durham, USA

## Purpose

We aimed to examine the prevalence of botanical dietary supplement (BDS) use among Hispanics in the United States (US) through a systematic review of the literature.

## Methods

We performed a systematic review of BDS use among adult Hispanics living in the US. Our strategy included electronic database searches (CINAHL, EMBASE, Global Health, CAB Abstracts, and Medline) with keywords: herbal, herb, medicinal plant, botanical and Hispanic or Latino along with a manual search of retrieved references. We included only studies with at least 1% Hispanics, prevalence estimates for Hispanics, and publication dates 1998-2011. We extracted information on study and sample characteristics, and rates of disclosures to clinicians.

## Results

Of the 35 studies reporting prevalence of BDS use among Hispanics, estimates ranged from 5% to 94% with a slightly narrower range restricting to use over the past 12 months (7-75%). Eighty-one percent (n=27) of studies included non-Hispanics and 54% reported <500 subjects. Smaller study size, regional location, convenience sampling, and longer recall periods were associated with greater prevalence of BDS use among Hispanics (Chi square, Fisher’s exact p<0.05). Studies with predominantly Hispanic samples also reported higher prevalence (p = 0.02). Of the predominantly Hispanic studies (n=11), 64% were conducted in states bordering Mexico, all in both English and Spanish. Rates of use and disclosure to clinicians ranged from 7 to 65% and did not depend on the presence of underlying health conditions. Health conditions included breast cancer, HIV, diabetes, heart disease, menopause, and pregnancy.

## Conclusion

Variability in reported prevalence of herb use among Hispanics depends, in part, on sample composition, possibly related to the use of Hispanic-specific instruments. Disclosure rates also varied widely, even in high risk illness. Because of marked variability in estimates, more research is needed to understand patterns of BDS use and disclosure among Hispanic populations in the US.

